# Harmony of Mind and Motion: Exploring the Interplay between Physical Activity and Mild Behavioral Impairment in Dementia‐Free Older Adults

**DOI:** 10.1002/alz.091908

**Published:** 2025-01-09

**Authors:** Dinithi M Mudalige, Dylan X. Guan, Eric E. Smith, Zahinoor Ismail

**Affiliations:** ^1^ University of Calgary, Calgary, AB Canada; ^2^ Hotchkiss Brain Institute, University of Calgary, Calgary, AB Canada; ^3^ Department of Clinical Neurosciences and Hotchkiss Brain Institute, University of Calgary, Calgary, AB Canada

## Abstract

**Background:**

While dementia typically presents in later life, early‐to‐mid‐life factors like physical inactivity contribute to dementia risk. Mild Behavioral Impairment (MBI), marked by emergent and persistent behavioral changes in older adults, also contributes to dementia risk. However, there is limited research on the impact of physical activity in those with MBI. In a community‐dwelling sample of dementia‐free older adults, we determined the association between the total weekly frequency of engaging in physical activity and MBI severity and whether this association depended on the total duration of physical activity.

**Methods:**

Data were obtained from the Canadian Platform for Research Online to Investigate Health, Quality of Life, Cognition, Behavior, Function, and Caregiving in Aging (CAN‐PROTECT) study. Measures included the MBI Checklist (MBI‐C) and physical activity questions from the Community Healthy Activities Model Program for Seniors (CHAMPS) questionnaire. Four physical activity domains were established (physical labor, cardiovascular activities, mind‐body exercises, and strength training). Multivariable negative binomial regression models modeled associations between MBI and weekly frequency of engaging in total and domain‐specific physical activity. All models were adjusted for age, sex, years of education, marital status, ethnocultural origin, and occupation type. Duration engaging in physical activity was explored as a covariate and a moderator.

**Results:**

For every 1 standard deviation (SD) increase in the total physical activity frequency was associated with a 18.3% (95%CI: ‐31.3—6.6, p = 0.002) lower MBI‐C total score. This association was moderated by total duration engaging in physical activity (expB = 1.0, 95%CI: 1.0‐1.0, p = 0.008), where the strength of the association weakened as the duration of physical activity increased. Additionally, every 1 SD increase in cardiovascular activity frequency and mind‐body activity frequency were associated with 18.1% (95%CI:‐30.9—6.5, p = 0.003) and 15.7% (95%CI:‐28.9—3.7, p = 0.006) lower MBI‐C total scores.

**Conclusions:**

More frequent weekly physical activity and cardiovascular and mind‐body exercise were associated with lower MBI severity. As the duration of physical activity increased, the impact of the frequency of physical activity on MBI severity decreased, and vice versa. Since lower MBI scores are associated with a lower risk for dementia, these results might inform dementia risk reduction, even in those with few cognitive symptoms.

